# NLRP12- associated autoinflammatory disorder: case report

**DOI:** 10.1186/1546-0096-12-S1-P262

**Published:** 2014-09-17

**Authors:** Giuseppina Volpe, Angela Mauro, Antonio Mellos

**Affiliations:** 1 ASL NA3 SUD, Terzigno, Italy; 2 Pediatric, Second University of Naples, Naples, Italy

## Introduction

During latest years, the identification of genes involved in the control of inflammation, apoptosis processes and a better comprehension of the mechanisms connected to an anomalous activation of the inflammasome, have made possible to delineate a new group of illness called " Monogenic autoinflammatory syndromes".

## Objectives

Report a case of recurring fever due to NLRP12 mutation.

## Methods

E.M., 4 years old, comes to our observation because of the presence of recurrent episodes of fever lasting 4-5 days,since about one year, every 15 days, associated with diarrhea, urticaria-like rush decreasing spontaneously, mainly on the back and lower limbs.

From November 2012 to April, the patient presented daily fever (Tmax 40°C) associated with worsening rush, arthralgia and arthritis of knees and ankles , edema of eyelids and lips and palmar angioedema during 7 days. From April 2013, every 15 days, the child had again febrile episodes (Tmax 40°C) lasting about seven days with the associated symptoms already described. Each episode has been exclusively cured with Paracetamol. Blood tests revealed just a small increase of the inflammatory indexes. After excluding other common causes of persistent and recurrent fever in the pediatric age, we suspected an autoinflammatory disease. Then , we directed the patient to a third level structure, where analysis of the CIAS1 gene have been carried out. Suspecting strongly an auto-inflammatory disease, the NLRP12 gene has been examined too. Analysis of the CIAS1 gene didn’t reveal pathological mutations, while the analysis of NLRP12 gene resulted positive.

## Results

Considering the clinical feature associated with increase of inflammatory indexes and the genetic analysis outcomes, the diagnosis of disease was NLRP-12 associated autoinflammatory disorder (NLRP12AD).

## Conclusion

The NLRP12AD is an autosomic dominant disease , due to mutation of NLRP12 gene which encode for the NLRP12 protein or "MONARCH-1", playing a crucial role in the immune mechanisms against pathogens agents. As for Cryopyrinopathies, symptoms can be inducted by the exposure to the cold and are characterized by recurring fever episodes lasting 5-10 days, associated with rash, headache, lymphadenopathies, oral ulcers and abdominal pain. Therapy depends on the seriousness of the symptoms: in less serious cases , treatement is based on antihistamines, NSAIDs and corticosteroids; in more serious ones, the administration of Anakinra can be useful. In the refractory cases, further therapeutic strategies are based on Anti-TNF and ANTI-IL-6 agents.

## Disclosure of interest

None declared.

